# L'Hopital De La Charite De Paris

**Published:** 1913-01-18

**Authors:** 


### L'HOPITAL DE LA CHARITE DE PARIS.

### II. ITS FURTHER ADVENTURES IN HISTORY.

At the commencement of this period the hospital possessed 216 beds, of which 51 were supported from ordinary revenue and 165 by special endowments. In 1790, Antoine, architect of La Monnaie, added a wing to the existing buildings. The brotherhood, despite the decree of 1789, continued to staff the hospital, but the disturbed times led to a considerable falling-off in their revenues. In addition, the newly-imposed land taxes hit the establishment hard. In one year the brothers had to pay a tax of no less than 1,454 livres on a single farm, despite the fact that all farm produce was consumed on the premises and none sold. Again, the municipality of Montevrain profited by the decree dissolving the religious orders to seize the crops from the land belonging to the hospital in its district. Moreover, organised begging was stopped, which again meant a reduction of revenue. The Government did its best to put these things to rights, but the great distress which prevailed on all sides gave the hospitals much extra work, with which they were unable to cope successfully The lack of an adequate number of female beds was severely felt. Accordingly in the year III. of the Republic, Clavareau, the architect of the hospitals, was commissioned to draw up a scheme and plans to increase the number of beds in L'hospice de l'Unité to 500, half of which were to be devoted to female patients, as well as plans for the erection of a clinical school and hospital.

The work of construction was delayed, and it was not until 1799 that the new school was opened, and a series of splendid clinical lectures delivered there by Corvisart. The old chapel had been requisitioned as a lecture theatre, a school of anatomy was started, and a museum of natural history and a botanical garden provided. Meanwhile the brotherhood continued its ministrations, and it was not until 1801 that it finally took leave of the hospital. It is interesting to note that among those appointed in 1802 to administer the hospital under the new conditions were two former members of the order who had reverted to civil life, namely, the superintendent, M. Turquie, and the steward.

In 1802, after the departure of the monks, the quarters formerly occupied by them were converted into wards for the reception of female patients, thereby adding 100 beds to the institution and bringing the number up to 310. In 1812 the care of the patients was handed over to the Augustinian sisters, five of whom were sent from the Hôtel-Dieu, the mother-house. These were replaced in 1817 by the sisters of Saint-Vincent-de-Paul, who in turn gave way in 1837 to the Augustinian sisters. In 1888 the hospital was handed over to a lay staff, and the names of the wards changed, the names of saints being replaced by those of eminent scientists, physicians, and surgeons. From 1803 to 1847 the head of the hospital was an "agent de surveillance," but from the latter date the title has been changed to that of director. In the early nineteenth century the hospital staff numbered sixty-three, and admissions varied between 2,800 and 3,000 per annum, with a mortality rate of 1 in 7. During the century the accommodation of the hospital was increased from time to time, so that in 1861, as the result of a new frontage added to the rue des Saints-Pères (the ground floor of which was let out as shops), it contained 475 beds. Since then further additions have been made, and to-day there are 650 beds.

The topography of the institution at the present time is the same as it was in the eighteenth century, but the main entrance is now situated in the rue Jacob. On the left is the porter's lodge, and then comes the out-patient department, with a large waiting-hall, consulting-rooms, and electro-therapeutic and dental departments.

On the right of the main entrance are the administrative offices and that of the director. To the left, in the first courtyard, is the pharmacy, and to the right the shops let by the authorities and facing the rue des Saints-Pères, the quarters for the visiting staff and the internes in medicine. The entresol comprises on the left the quarters of the staff, on the right those of the porter and director's clerk, and then the shop buildings. The first floor is occupied on the right by the director's apartments, those of the steward and of the steward's clerk, on the left by the salle Velpeau and its amphitheatre, and in front finishing the quadrilateral by the salle Trélat. On the second floor, above the salle Velpeau, is the salle Andral, while above the Trélat is the salle Gosselin. On the right is the library, the rooms of the "internes," those reserved for the isolation of cases from the salle Trélat, an operating theatre, and the pharmacist's quarters. The top of the building is occupied by dormitories and rooms of the staff. In the second court the ground floor comprises to the left a portion of the pharmacy, the kitchen, and the rooms connected with it; in front the store-room of the kitchen, the steward's offices, and the store for the patient's clothing; to the right the continuation of the shops in the rue des Saints-Pères. On the first floor to the left is the salle Boyer, in front the salle Rayer, and to the right the salle Louis. On the next floor above Boyer is the salle Petit, above Rayer the salle Briquet, and above Louis, the crèche. In the third court on the ground floor to the left is another kitchen and rooms connected with it, the tailor's shop, the refectories, and the wash-houses; in front the linen-store and store-rooms; to the right the remaining shops of the rue des Saints-Pères. On the first floor to the left the salles Laënnec and Boulliand, to the right the salle Vulpian and a linen-store; in front a lecture theatre, rooms of the Academy of Medicine, and an employee's quarters. On the second floor to the left the salles Frère-Côme and Priory; to the right the salles Beau and Damaschino, and in front the salle Corvisart, the laboratories and rooms connected therewith. The top of the building is given over to two small wards, the salles Cruveilhier, and to an employee's quarters. In the second court to the left is an archway leading to the entrance to the main staircase. Continuing straight on, one comes to the baths, the stables, and coach-house, above which are a number of quarters for domestics. To the left after coming through the archway are the workshops, the chapel, the mortuary, and the post-mortem room. Behind and parallel to the chapel is a recreation ground for male patients and a store-house for bedding. In passing the kitchen buildings on the right and before coming to the plumber's workshop, which is at the end of the court, one passes through an alley and a last court on the left, which leads to the extremity of the hospital and the maternity department. In the main the men's wards are on the first floor, the women's on the second, and with one exception each visiting medical officer has his female immediately over his male wards. In 1898, 9,074 in-patients and 21,241 out-patients were treated in the establishment. In 1897 the money paid to run the hospital amounted to 777,600 francs, or £31,104.

